# Xeno-learning: knowledge transfer across species in deep learning-based spectral image analysis

**DOI:** 10.1038/s41551-025-01585-4

**Published:** 2026-01-26

**Authors:** Jan Sellner, Alexander Studier-Fischer, Ahmad Bin Qasim, Silvia Seidlitz, Nicholas Schreck, Minu Tizabi, Manuel Wiesenfarth, Annette Kopp-Schneider, Janne Heinecke, Jule Brandt, Samuel Knoedler, Caelan Max Haney, Gabriel Salg, Berkin Özdemir, Maximilian Dietrich, Maurice Stephan Michel, Felix Nickel, Karl-Friedrich Kowalewski, Lena Maier-Hein

**Affiliations:** 1 Division of Intelligent Medical Systems, German Cancer Research Center, Heidelberg, Germany; 2 Helmholtz Information and Data Science School for Health, Heidelberg/Karlsruhe, Germany; 3 Faculty of Mathematics and Computer Science, Heidelberg University, Heidelberg, Germany; 4 National Center for Tumor Diseases Heidelberg, a partnership between German Cancer Research Center and Heidelberg University Hospital, Heidelberg, Germany; 5 Department of General, Visceral, and Transplantation Surgery, Heidelberg University Hospital, Heidelberg, Germany; 6 Department of Urology and Urosurgery, Medical Faculty of the University of Heidelberg, University Medical Center Mannheim, Mannheim, Germany; 7 Division of Intelligent Systems and Robotics in Urology, German Cancer Research Center Heidelberg, Heidelberg, Germany; 8 DKFZ Hector Cancer Institute at the University Medical Center Mannheim, Mannheim, Germany; 9 Division of Biostatistics, German Cancer Research Center, Heidelberg, Germany; 10 Medical Faculty, Heidelberg University, Heidelberg, Germany; 11 Department of Anesthesiology, Medical Faculty, Heidelberg University Hospital, Heidelberg University, Heidelberg, Germany; 12 Department of General, Visceral and Thoracic Surgery, University Hospital Hamburg-Eppendorf, Hamburg, Germany

**Keywords:** Health care, Applied optics, Computational science, Translational research

## Abstract

Optical imaging techniques, such as hyperspectral imaging combined with machine learning-based analysis, have the potential to revolutionize clinical surgical imaging. However, these modalities face a shortage of large-scale, representative clinical data for training machine learning-based algorithms. While preclinical animal data are abundantly available through standardized experiments and allow for controlled induction of pathological tissue states, it is not ethically possible to obtain similar data from patients. To leverage this situation, we propose ‘xeno-learning’, a cross-species knowledge-transfer concept inspired by xeno-transplantation. Here, using a total of 14,013 hyperspectral images from humans as well as porcine and rat models, we show that, although spectral signatures of organs differ substantially across species, relative changes resulting from pathologies or surgical manipulation such as malperfusion or injection of contrast agent are comparable. Such changes learnt in one species can be transferred to a new species through a ‘physiology-based data augmentation’ method, enabling the large-scale secondary use of preclinical animal data for human application. The resulting benefits promise a high impact of the proposed knowledge-transfer concept on future developments in the field.

## Abstract (web summary)

Xeno-learning uses preclinical data from one species to boost the performance of neural networks applied to another species when exploring physiological responses.

## Main

One of the major challenges faced by surgeons is the visual discrimination of tissues, for example, to distinguish between pathological and physiological tissue or spare critical intraoperative structures. Spectral imaging has been proposed as a means of overcoming the limitations of visual perception^1^. While conventional medical cameras (for example, laparoscopic imaging systems) are limited by ‘imitating’ the human eye and recording only red, green and blue colours, spectral cameras remove this arbitrary restriction and instead capture multiple specific bands of light that allow for decoding relevant information on tissue type and function^2,3^.

Artificial intelligence (AI) in general and machine learning (ML) in particular have evolved as key enabling techniques to convert the high-dimensional spectral data into clinically useful information^4–7^. However, a major bottleneck in converting this imaging technique’s potential into patient benefit lies in the lack of large annotated, high-quality datasets covering the wide range of pathologies that can occur in practice. In this context, preclinical animal data represent an untapped resource, offering not only more data but also the possibility of conducting various types of standardized experiments. This is in stark contrast to human data acquisition for which high ethical standards and clinical workflow considerations render many experiments of high scientific interest infeasible, for example, the intentional induction of pathological tissue states such as fibrosis, inflammation or malperfusion.

**Fig. 1:**
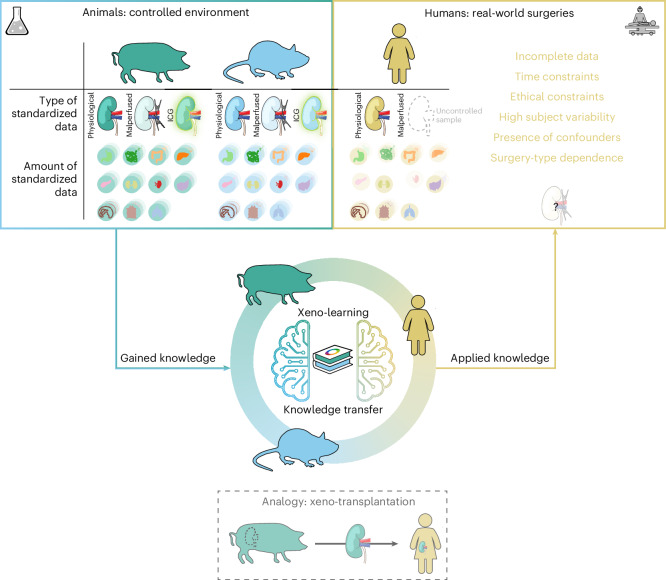
Inspired by the concept of xeno-transplantation, transplanting an organ from one species to another, we propose xeno-learning as a concept for transferring knowledge across species. Human data obtained with novel imaging modalities, such as HSI, are often sparse, do not represent the broad range of pathologies or surgical manipulations (for example, malperfusion and injection of contrast agent) that can occur in practice, and lack standardization owing to ethical and clinical workflow issues. We introduce the method of xeno-learning as a concept to boost the performance of neural networks applied to one species by making efficient use of data from another species. Organ icons adapted from ref. ^2^, CC BY 4.0.

**Fig. 2:**
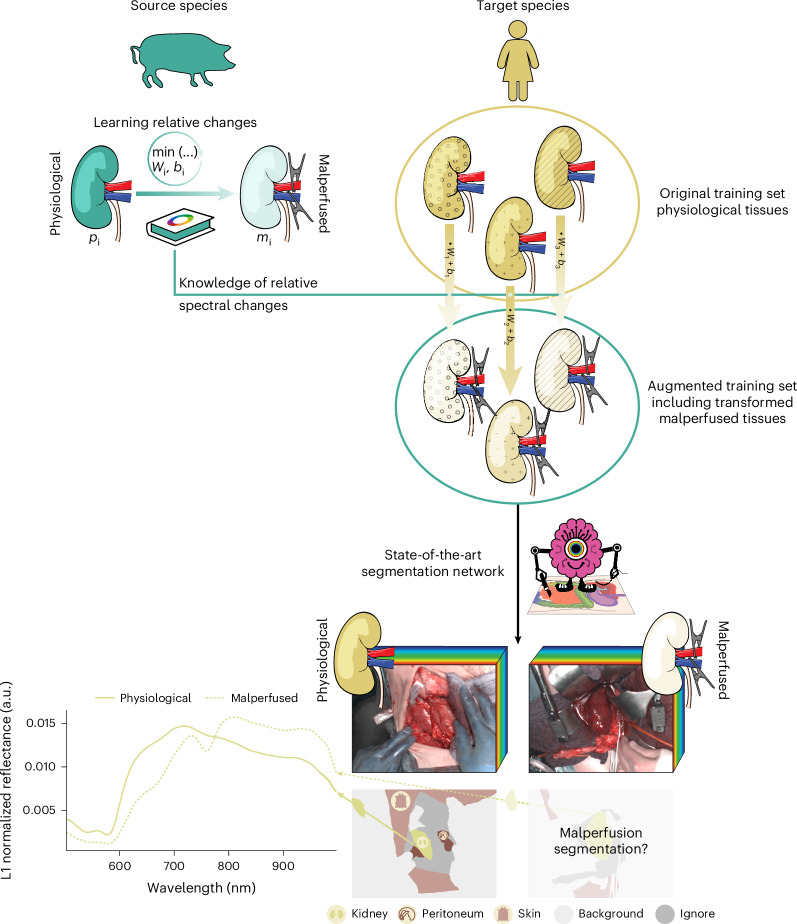
Knowledge on perfusion shifts is transferred via a physiology-based data augmentation method. The example shows how relative spectral changes resulting from perfusion shifts are learnt from physiological and malperfused tissue pairs (*p*_i_*, m*_i_) of the source species and encoded in a linear model. The target species’ training set, which contains only physiological tissues, is augmented by applying the learnt model to the target spectra. State-of-the-art segmentation networks^8–10^ are applied for surgical scene segmentation. The example images show human physiological and malperfused kidneys, their corresponding semantic segmentation and median spectra for the annotated kidney regions. The example focuses on pig as the source and human as the target species, with malperfusion as data shift. However, the same method can also be applied to other species or data shifts, such as those arising from the application of contrast agents, for example, indocyanine green (ICG). Organ icons adapted from ref. ^2^, CC BY 4.0. Hyperspectral tissue classification (HTC) icon adapted from ref. ^8^, CC BY 4.0.

To address the human data bottleneck, we therefore propose the concept of ‘xeno-learning’ (Fig. 1), an AI method for transferring knowledge across species. The term has been inspired by the concept of xeno-transplantation, which refers to the transplantation of living cells, tissues or organs from one species to another. In this case, the shortage of transplantable human organs is addressed by using organs from other species; in analogy, our work aims to address the shortage of standardized data by transferring knowledge from one species (specifically porcine or rat models) to another (here, humans). The following are our specific contributions. (1) Knowledge-transfer bottleneck: We demonstrate that spectral signatures of organs differ across species, thus causing neural networks trained on one species (for example, animals) to fail when classifying tissues of another species (for example, human tissue). (2) New cross-species learning approach: We introduce the concept of xeno-learning as a method to boost the performance of neural networks applied to one species by making efficient use of data from another species. We further instantiate the concept on the specific challenge of perfusion shifts in spectral image analysis, which may lead to radical performance decrease in state-of-the-art tissue segmentation methods. Relative spectral changes are learnt in one species and transferred to another species using a ‘physiology-based data augmentation’ method (Fig. 2). (3) Comprehensive validation with three species: On the basis of a hyperspectral imaging (HSI) database comprising 14,013 hyperspectral images from humans as well as porcine and rat models, we show that tissue discrimination performance can be boosted by learning from another species.

## Results

All of the presented results were obtained using the medical device-graded HSI system Tivita Surgery (Diaspective Vision) to collect 14,013 HSI images from three species including 230 patients from Heidelberg University Hospital (Supplementary Fig. 1). For our analysis, we semantically annotated 2,596 images with 12 classes (11 organ classes and background).

### Differences in organ spectra result in segmentation network failure to generalize across species

**Fig. 3:**
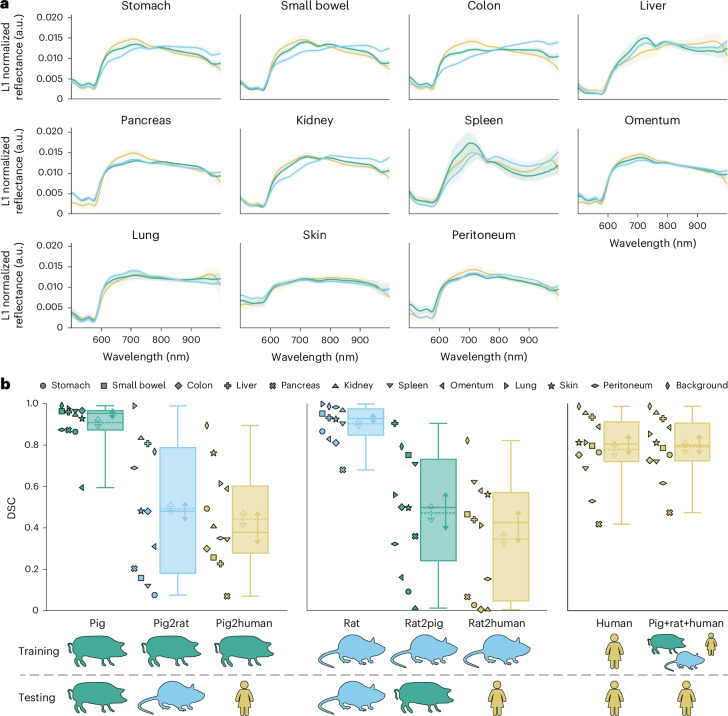
Different species exhibit different spectral organ fingerprints, resulting in organ segmentation networks trained on one species failing to generalize towards unseen species. **a**, For each organ and species, the median spectrum (solid line) and the s.d. (shaded area) across subjects are shown. The median spectrum was calculated per channel for each annotated region in all images. **b**, For each species, the intraspecies segmentation performance always surpasses the out-of-species performance. The distributions of hierarchically aggregated class-level DSC scores are shown (*n* = 12 classes). Each box plot depicts the interquartile range (IQR) with the median (solid line) and mean (dotted line). Arrows indicate the 95% confidence interval of the median and mean based on bootstrapped subject sampling. The whiskers extend up to 1.5 times the IQR. Results for the NSD are shown in Supplementary Fig. 2. Pig2human, pig to human; Pig2rat, pig to rat; Rat2human, rat to human; Rat2pig, rat to pig.

While similarities do exist, spectral organ fingerprints were generally not consistent across species. Figure 3a shows spectral organ fingerprints for a total of 11 organs for three species, namely, humans, pigs and rats. Some organs, such as the skin or omentum, exhibited higher similarities in their spectra across species than other organs did, such as the colon or kidney.

To investigate the effect of the spectral differences on neural network performance, we trained networks on physiological organ images of one species and evaluated their performance on physiological organ images of other species (Fig. 3b).

Segmentation networks failed dramatically when applied to a new species, with performance decreases of −43% (pig to human) to −56% (rat to human). The metric values also varied highly between different classes: whereas classes such as background or skin could still be detected correctly in many cases, other classes such as pancreas exhibited high performance drops. Joint training of animal and human data (Fig. 3b, pig+rat+human network) did not improve the discrimination of human tissues.

It should be noted that the intraspecies performance values (Dice similarity coefficient (DSC), Fig. 3b; normalized surface Dice (NSD), Supplementary Fig. 2) are consistent with state-of-the-art literature^8–10^. Overall, the segmentation performance on human data was lower (0.78 (standard deviation (s.d.) 0.17)) than on animal data (0.91 (s.d. 0.11) and 0.90 (s.d. 0.09) for pig and rat, respectively).

### Extended data analysis provides reasons for neural network failure

To quantify the relevance of the species relative to other sources of variation, such as the subject or the specific imaging conditions, we performed a mixed effect model analysis. For both pigs and rats, we followed the standardized recording protocol defined in the previous data publication^11^, according to which spectral images from multiple subjects are acquired for a predefined set of recording conditions. For each organ, the proportion of variation in observed reflectance was decomposed into (explained) variation by the factors species, subject, image and angle, as well as residual or unexplained variation, as shown in Supplementary Fig. 3.

To facilitate knowledge transfer, a negligible species effect would be desired. However, this is not the case, with no consistent behaviour to be observed for all organs. For several organs, the species even constituted the main source of variability for certain wavelengths. Across all organs, the angle factor and unexplained variation (residuals) only played a minor role.

**Fig. 4:**
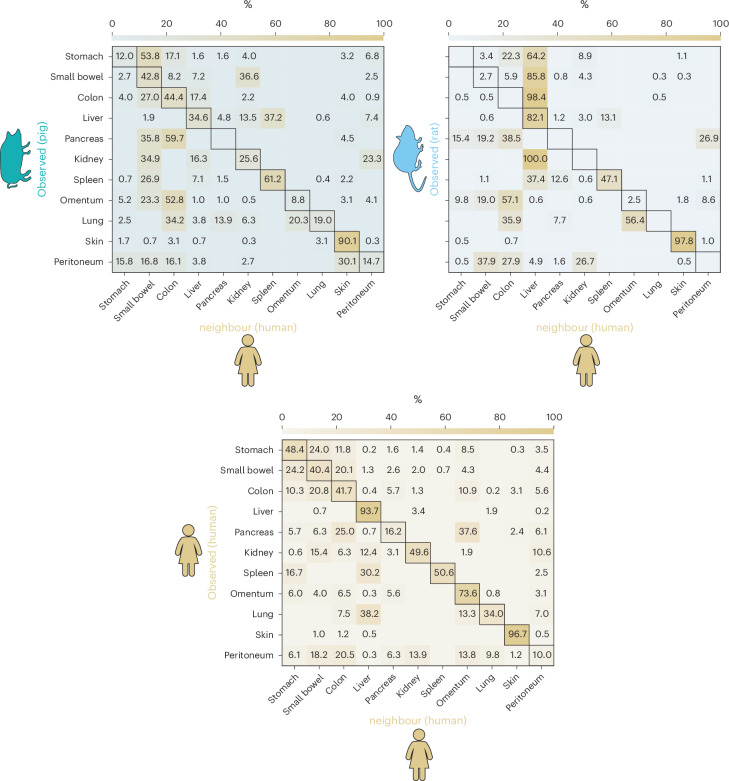
Spectra of the same tissue type are not nearest neighbours across species. Comparison of median spectra per organ between pig and human (top left), rat and human (top right) and human to human participants (bottom left). For every median spectrum of the observed species, we determined the nearest neighbour in the human species and compared the class labels. The nearest neighbour spectrum is always from a different subject. Each matrix is row normalized, highlighting how the median spectra from an animal class are distributed across the nearest neighbour human classes. Animal comparisons are shown in Supplementary Fig. 4.

Besides the variability analysis of the spectra, we performed an analysis to examine the extent to which spectral values matched between organs. Figure 4 and Supplementary Fig. 4 accordingly show the high-dimensional neighbourhood of the median spectra. When comparing organ spectra of one species to those of another, it could be observed that the nearest neighbour only rarely agreed in the organ class (low agreement on the diagonal). For example, porcine spectra of the pancreas were closer to human colon or small bowel spectra than human pancreas spectra. Noticeable exceptions were liver spectra and skin spectra, which were relatively close to each other for all species. By contrast, the nearest neighbours between different human participants showed a much higher agreement.

### Shared mechanisms lead to comparable relative spectral changes across species

Our previous analyses clearly showed that the substantial absolute differences of spectral organ signatures across species render naive approaches of knowledge transfer infeasible. Consequently, we investigated whether relative changes in spectra resulting from pathologies (for example, ischaemia) or surgical manipulation (for example, injection of contrast agent) exhibited similarities across species. We focused on tissue malperfusion, an essential aspect of real-world surgeries, as well as spectral changes following intravenous ICG injection, an important mechanism for clinical, intraoperative perfusion monitoring. These changes may result in poor tissue discrimination performance for neural networks trained primarily on physiological tissue. The underlying hypothesis was that the comparable tissue alterations result in similar spectral changes in humans and animals.

**Fig. 5:**
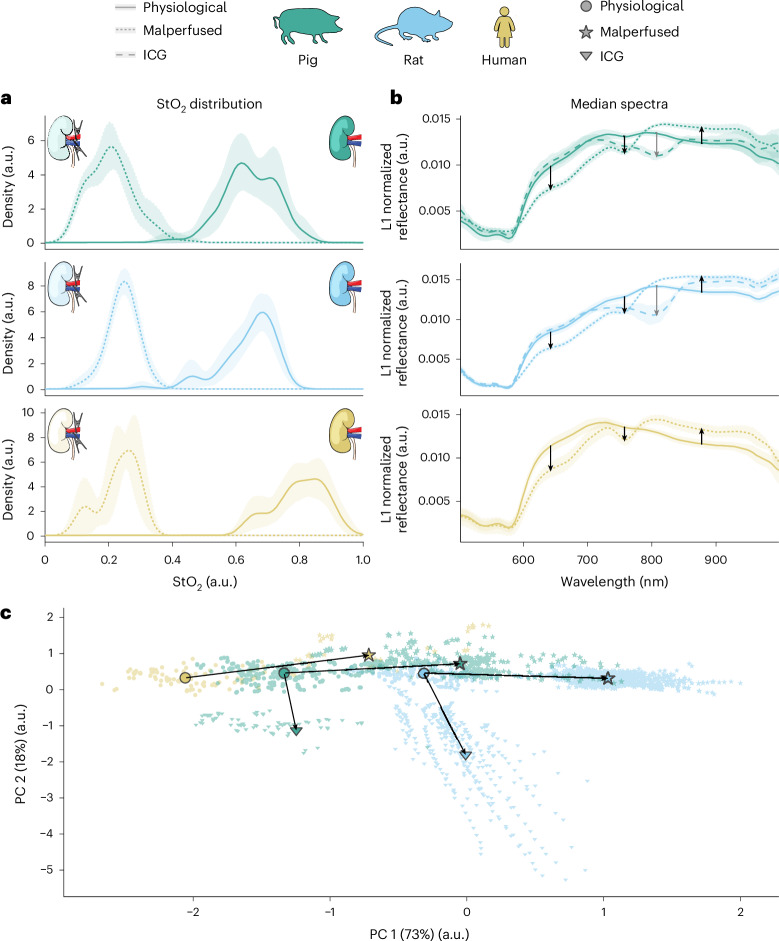
Although spectra are different across species, pathologies or surgical manipulations lead to comparable systematic spectral shifts. **a**,**b**, For each species, the distribution of tissue oxygen saturation (StO_2_) for physiological and malperfused kidney tissues is shown (**a**). The corresponding median spectra are shown, highlighting transitions from physiological to malperfused states and shifts following ICG injection (**b**). The shaded area highlights the 95% confidence interval based on bootstrapped subject sampling (StO_2_ distribution) and the s.d. across subjects (median spectra). **c**, PCA of the median spectra indicates a spectral shift in the same direction across species when transitioning from physiological to malperfused tissues (in this case, to the right) or from physiological to ICG-injected spectra (in this case, to the bottom). Solid markers denote the cluster centres, and each transparent marker represents one image, whereas the axis labels denote the explained variance of the corresponding principal component.

We thus systematically compared physiological and malperfused organ spectra as well as spectra following ICG injection (Fig. 5). To this end, we focused on kidneys, as they provide a highly standardized, biologically unambiguous and clinically relevant model. Although the spectra differed, the changes from physiological to malperfused spectra or the changes from physiological to spectra following ICG injection exhibited a similar pattern across all species. For example, a drop in normalized reflectance could consistently be observed around 750 nm in the case of malperfusion. Low-dimensional projections of the kidney median spectra further illustrate that despite the only minor overlap of physiological samples between species, a similar shift can be observed when transitioning from physiological to malperfused tissue or from physiological to ICG-injected tissue in principal component analysis (PCA) space.

### Xeno-learning enables knowledge transfer

Our approach to knowledge transfer is based on the crucial insight that shared mechanisms manifest in comparable relative spectral changes. As shown in Fig. 2, we leverage this insight through data augmentation; we first learn the effect of a pathology or intervention from data of the source species and then apply this knowledge to the target species. By analogy with physics-based data augmentation, we termed this method ‘physiology-based data augmentation’.

**Fig. 6:**
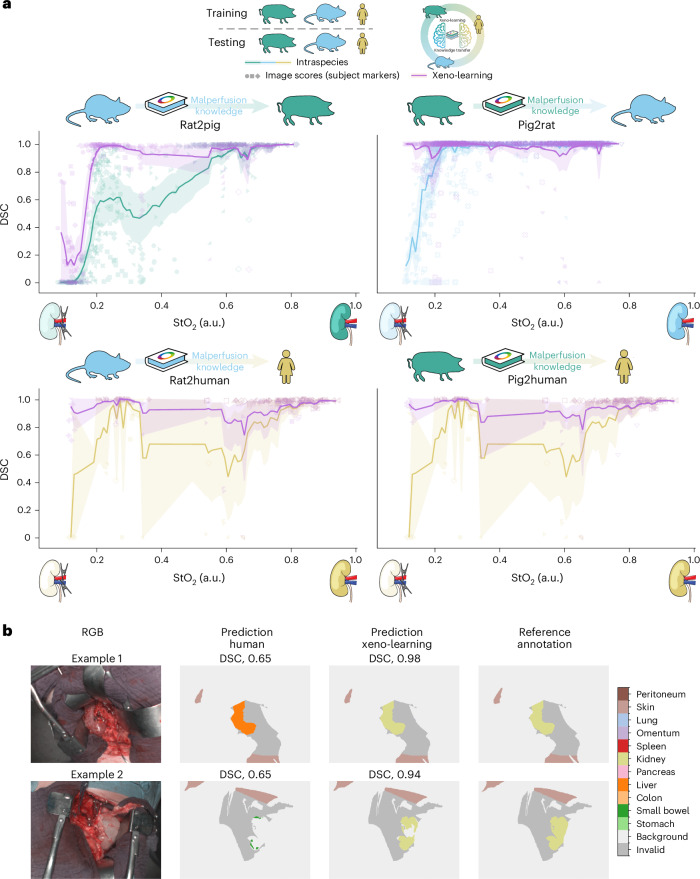
Xeno-learning enables knowledge transfer across species. **a**, The segmentation performance, measured via the DSC, is substantially improved with the xeno-learning approach, especially for malperfused tissue corresponding to low StO_2_. For each subject in the target species, a line was interpolated over all (StO_2_ and DSC) values and hierarchically aggregated across subjects. The shaded area denotes the 95% confidence interval based on bootstrapped subject-sampling, and the transparent markers denote the raw kidney scores for individual images (different markers correspond to different subjects). **b**, The human baseline network fails to correctly classify the kidneys in the example images, while the pig2human network, which includes our xeno-learning transformation, segments the kidneys with high accuracy.

According to experimental results obtained on independent test sets that were not used during method development, our proposed xeno-learning method can transfer relevant perfusion-related knowledge encoded in preclinical animal data to humans (Fig. 6).

**Fig. 7:**
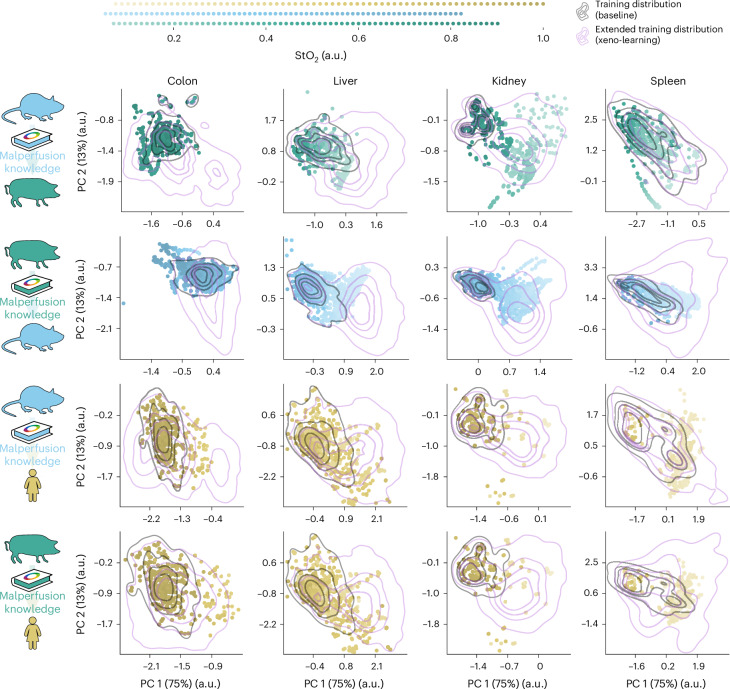
Xeno-learning extrapolates the training data to cover a wide range of spectral perfusion states for multiple organs. PCA of all median spectra from the target species are shown as circles with the opacity encoding the StO_2_ of the tissue. The training distribution of the baseline as well as the extended training distribution obtained via our physiologically based augmentation was estimated by kernel density estimations and is depicted with grey (original) and purple (xeno-learning) lines. For many organs and species, malperfused spectral data are out of distribution with respect to the original training distribution. Xeno-learning resolves this issue.

As shown in Fig. 7, the original training data (distribution depicted in grey) do not adequately cover the malperfused data (green, blue and yellow points depending on species). Xeno-learning (distribution coloured in purple) resolved the issue. To quantify the effect of our augmentation on the training distribution, we calculated the distances between each point in the test dataset to the nearest point in the training dataset. We observed that the distances (averaged over all organs) decreased by 29.7% (s.d. 12%) for the rat2pig, 27.6% (s.d. 12%) for the pig2rat, 26.5% (s.d. 8%) for the rat2human and 20.5% (s.d. 6%) for the pig2human scenarios when evaluated against the extended training distribution as opposed to the baseline training distribution.

Whether or not a change in spectra resulting from pathophysiological effects results in a drop in the performance of the downstream tasks depends crucially on the specific application. In the case of semantic segmentation, the only major drop in performance was observed for the kidney (Fig. 6). In general, malperfused tissues present a challenge to the segmentation network across all species and the DSC tends to decrease with lower StO_2_. In the case of humans, the knowledge transfer was more beneficial than in rats.

### Xeno-learning generalizes across knowledge-transfer tasks

To demonstrate that xeno-learning generalizes over knowledge-transfer tasks, we prospectively applied our approach to a more complex pathophysiological mechanism without making any changes to the method. Specifically, we investigated whether spectral changes resulting from the injection of the contrast agent ICG can be learnt across species. By analogy with our first knowledge-transfer task, we encoded relative spectral changes from data without contrast agent to data with ICG and transferred them to another species via data augmentation.

**Fig. 8:**
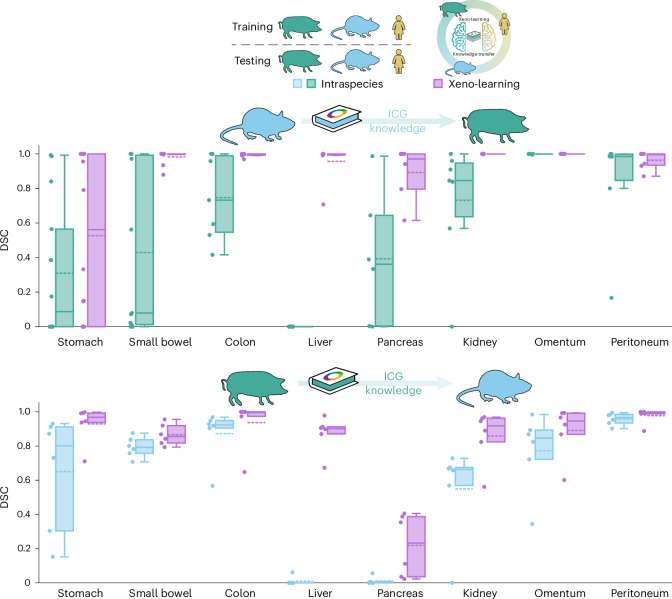
Xeno-learning recovers segmentation performance after ICG fluorescence injection across organs. While neural networks trained on data without contrast agent fail to generalize to data with contrast agent (green and blue plots), xeno-learning compensates for this issue (purple plots). The segmentation performance was measured via the DSC for *n* = 15 pig and *n* = 6 rat subject-level scores. Each box plot depicts the IQR with the median (solid line) and mean (dotted line). The whiskers extend up to 1.5 times the IQR. Every point represents the segmentation performance of one subject and organ.

As shown in Fig. 8, neural networks trained on data without contrast agent fail to generalize to data with contrast agent. This holds true across species and organs. In all cases, our xeno-learning method could recover the segmentation performance. For the porcine data, our approach led to an average increase of the DSC of 0.36 (s.d. 0.26), with the liver showing the biggest improvement of 0.96. Similarly, for the rat data, we obtained an average increase in DSC of 0.24 (s.d. 0.17), with the liver showing the biggest improvement of 0.86.

## Discussion

With this systematic, in-depth analysis of spectral organ differences across species, we demonstrate that spectral knowledge transfer across species is possible, as exemplified by the task of surgical scene segmentation and the transfer of different perfusion or ICG states. On the basis of a large HSI database containing 14,013 images from 319 subjects in three species from which 2,596 images have been fully semantically annotated with 12 classes, we derived the following key findings. (1) Inter-species differences of organ spectra are substantial: Different organs across species do not display uniform spectral organ fingerprint features. When considering the species, the subject and imaging-related factors as sources of spectral variability, the species even represents the main source of variability for certain wavelengths and organs. This heterogeneity causes state-of-the-art segmentation networks to fail when applied across species. (2) Shared mechanisms manifest as comparable relative spectral changes across species: Substantial absolute differences of spectral organ signatures across species render naive approaches of knowledge transfer infeasible. However, our study revealed that relative changes in spectra resulting from pathologies or ICG-affected organs exhibit similarities across species. (3) Xeno-learning enables knowledge transfer across species: A proof-of-concept study revealed that the proposed concept of learning across species to compensate for quantitative and qualitative data shortage is feasible. From a methodological perspective, the enabling idea was to transfer relative spectral changes via a means of physiological data augmentation.

### Strengths and limitations of the method

One of our key observations is that organ differences are pronounced between species and that segmentation networks struggle when applied to another species. Despite that fact, our proposed xeno-learning method can still improve the segmentation on malperfused and ICG-affected spectra. A key to this success lies in the fact that our xeno-learning method is independent of the absolute organ differences between the species, as it captures the relative change from a physiological to a malperfused or ICG-affected organ.

In this proof-of-concept study, we model relative spectral changes resulting from pathologies or surgical manipulation states via a linear transformation which is applied separately to each pixel in the image and which consists only of a manageable number of learnable parameters (100 *×* 100 + 100 = 10,100 per image pair). This restriction naturally reduces the risk of overfitting, as there is less freedom to account for unwanted side effects (for example, from outliers in the data) during the optimization process of the transformation. Although simple by design, it has proven to be a powerful tool to model perfusion changes. Its linearity substantially facilitates computation (affine operation), with only marginal computational overhead during training. However, these restrictions have the disadvantage that the transformation cannot learn nonlinear changes. Furthermore, several applications may require the learning of texture changes and thus require the method to be expanded to larger patches. More specifically, by replacing pixel-wise transformations with patch-wise transformations, it could be possible to alter the texture of tissues using image-to-image translation techniques. However, to keep the computational complexity manageable, it may be necessary to use relatively small models.

Our experiments in Fig. 5 clearly demonstrate that malperfused organs are out of distribution with respect to the physiological training data. While this effect did not lead to a radical performance drop for the specific downstream task of semantic segmentation for all organs, our analyses show that the augmented data capture the malperfused data substantially better.

### Strengths and limitations of the study

The fact that spectra differ across species is not surprising, as the basis of spectral tissue properties is the biomolecular composition of the organism, which is highly different between species^12^. The most widely studied and spectrally influential protein is haemoglobin, which is known to be built differently between species, hence exhibiting different spectral properties under varying environmental conditions and affecting organs differently^13,14^. Analogous principles apply for other chromophores such as melanin, myoglobin or bilirubin^15^. At the same time, it is also known that there are shared pathophysiological mechanisms across species. This includes the characteristic changes on the absorbance curve of haemoglobin^16–18^. In this Article, we showed that such shared pathophysiological mechanisms manifest as comparable relative spectral changes across species, which can then be used for xeno-learning.

While we extensively analysed the spectral characteristics of 11 physiological organs, we restricted our xeno-learning approach to two exemplary scenarios: correcting the segmentation of kidneys despite different perfusion states (physiological and malperfused) and fluorescent dye application. This decision was made for two main reasons: first, kidneys offer a very standardized and controlled setting for inducing pathological organ states through their singular hilar blood supply by clamping or contrast agent application; and second, these are relevant clinical states regularly encountered during kidney tumour surgery or kidney transplantation. In addition, kidney pathology has been of interest in other studies on differentiating arterial ischaemia and venous congestion^19^, assessing kidney function in human kidney allotransplantation^20^ or monitoring oxygen saturation during normothermic machine perfusion^21^.

Generally, opportunities for systematic and standardized HSI data acquisition are highly limited in humans for ethical and regulatory reasons. Furthermore, human data rarely capture spectral changes in isolation without the influence of other pathologies (for example, thrombosis), surgical interventions (for example, cauterization) or side effects from surgical procedures such as kidney transplantations (for example, medication or host-versus-graft reactions). Organ surfaces may also be less exposed than in animal experiments, such as in cases of adherent renal fat. All these factors make human data acquisition and processing especially challenging. They may also explain the generally lower segmentation performance of neural networks on human data shown in Fig. 3b. We addressed the human data acquisition issues with xeno-learning. The animal malperfusion states and ICG data, induced with high standardization by hilar clamping and systemic intravenous ICG application, possess a high temporal resolution, as evidenced by the number of image data points in Fig. 6. Knowledge acquired through these systematic experiments has been successfully transferred to a human application.

### Our method in the context of domain adaptation

Our method can be classified as a domain adaptation technique, as it leverages data from both the source and target domains to enhance performance in the target domain^22^. It is specifically designed for cross-species knowledge transfer in the context of spectral data. We intentionally distinguish between the information we aim to transfer—relative changes in the spectra—while explicitly avoiding the transfer of other types of information, such as absolute spectral reflectance values which are known to be different for each species (Fig. 5). This is in contrast to domain adaptation methods that directly make use of the absolute spectral values together with the domain information. A comparison of our method with a domain adaptation technique is shown in Supplementary Fig. 5.

Unlike other domain adaptation methods, our approach promotes data sharing without requiring access to the source data, as only the learnt relative differences are needed. For example, we provide the necessary projection matrices for applying our physiologically based data augmentation, enabling anyone to use our method on their spectral data.

### Comparison with other (multispecies) studies

Previous studies which included multiple species focused purely on spectral analysis without the aim to transfer knowledge in the context of AI training. For instance, different publications compared spectra from humans and pigs with a focus on the gastric conduit^23^, kidney perfusion states^19^ and on organs of the biliary system^24^, respectively. One^23^ included gastric conduit data from ten patients and found comparable StO_2_ values across species for the malperfusion subgroup. The kidney results from 17 patients indicate similarities in the malperfusion spectra^19^, in line with our results. The presented data partly overlap with our data. While only healthy organs of the biliary system from the seven patients were considered^24^, relevant spectral differences between humans and pigs could be observed, further supporting our findings. It should be noted that these works, however, used substantially smaller sample sizes in the range of 7–17 patients, while our cohort included 230 patients. Furthermore, none of them used data from one species for an application to another species.

In the domain of HSI, surgical scene segmentation with fully semantically annotated HSI has, until now, been limited to porcine models. The largest study^9,10^ so far comprises 600 images from 33 pigs annotated with 19 classes, with the data partly overlapping with our data. The reported DSC and NSD scores are in agreement with our porcine and rat models (Fig. 3b and Supplementary Fig. 2). Results and solutions regarding the robustness of HSI models towards geometrical domain shifts in this study were incorporated into our models.

Human tissue recognition in general has been performed ex vivo and in vivo^25^. The largest study is from the EX-MACHYNA trial^26^ involving 169 patients undergoing elective open abdominal surgery from two medical centres annotated with 13 tissue classes. While annotations are not fully semantic, they closely follow organ boundaries. The reported DSC of 0.80 is in agreement with our reported DSC of 0.78 (s.d. 0.17) on human data (Fig. 3b). Here, the main differences from our study lie in the fact that we included malperfused and ICG data as well as data from three species and explicitly leverage animal data to make the segmentation performance of human data more robust.

Perfusion shifts in human kidney spectra have been analysed in previous works^27^. They monitored human patients undergoing partial nephrectomy using a video-rate multispectral imaging device to assess perfused and malperfused kidneys. Their low-dimensional projections, obtained via PCA, consistently demonstrated that the spectral shifts from physiological to malperfused kidneys followed a uniform direction. These findings are in strong concordance with our results shown in Fig. 5, suggesting that this behaviour is consistent across both multispectral and hyperspectral data, as well as across different species.

### Impact and future directions

While our xeno-learning concept primarily demonstrates the potential for enhanced generalizability across species, it also holds promise for the generalization of other variations. For instance, insights into disease models, various pathologies or imaging conditions observed in one species can potentially be transferred to others. One prominent example of a use case is cancer, a disease marked by differences in perfusion and oxygenation between physiological and pathological tissue. Overall, the expansion of xeno-learning to different use cases will require users to identify shared mechanisms across species and use that prior knowledge to enable the knowledge transfer for AI. Similarly, while our proposed augmentation method is primarily used for xeno-learning, its application is by no means limited to cross-species learning. From the perspective of transfer learning in AI systems, the method can be broadly used to identify variations within a source data distribution and subsequently integrate these insights during training within a target data distribution. For instance, observed tissue necrosis, tissue fibrosis or tissue inflammation in one population can be effectively applied to another population.

Notably, our xeno-learning method uses a simple linear model to transform between two spectral states—in our case, physiological and malperfused or ICG. Owing to its simplicity, this method could contribute to the understanding of such spectral changes, for example, by analysing the properties of our transformation matrices. This is especially crucial for human data, which is often confounded by therapy effects, comorbidities or surgical procedures not present in animal experiments^28,29^.

Our results can impact the planning of future animal studies and their potential to be applied to humans. For example, because the rat liver is closer to the human liver than to the pig liver, the rat model may be more appropriate for future studies targeting liver pathologies. By contrast, for other organs such as the pancreas, neither the pig nor the rat model would be an appropriate candidate.

### Conclusion

We show that knowledge transfer can be applied in the general context of AI-based image analysis. Our study with HSI data from three species demonstrated the potential of large-scale secondary use of preclinical animal data for humans. The resulting ethical, monetary and performance benefits of the proposed knowledge-transfer method promise a high impact of the methodology on future developments in the field. For maximum impact, our code and pretrained models will be made publicly available.

## Methods

### Data collection

The HSI animal data were collected at Heidelberg University Hospital following approval from the Committee on Animal Experimentation of the regional council Baden-Württemberg in Karlsruhe, Germany (approval nos. G-161/18, G-262/19 and G-62/23). The animals were treated in accordance with German laws for animal use and care, as well as the directives of the European Community Council (2010/63/EU).

The HSI human data were obtained during the SPACE trial (Spectral Characterization of Organs and Tissues during Surgery) at Heidelberg University Hospital, with approval from the Ethics Committee of the Medical Faculty of Heidelberg University, Germany (S-459/2020). The trial adhered to the ethical principles of the Declaration of Helsinki^30^ and the principles of Good Clinical Practice^31^. The trial’s reporting followed the recommendations of the Consolidated Standards of Reporting Trials (CONSORT) guideline^32^. The SPACE trial was registered with the Research Registry (researchregistry6281) on 23 November 2020.

### Hyperspectral image acquisition

The HSI camera system Tivita Tissue (Diaspective Vision GmbH) was used to collect the HSI data. This system captured hyperspectral images in a push-broom manner with a spectral resolution of approximately 5 nm, covering the spectral range from 500 nm to 1,000 nm. The resulting data cubes have dimensions of 640 × 480 × 100 (width in pixels × height in pixels × number of spectral channels). The camera system imaged an area of about 20 × 30 cm. An integrated distance calibration system, consisting of two light marks that overlap when the distance is correct, ensured an imaging distance of around 50 cm. The image acquisition process took approximately 7 s.

In addition to the HSI data cubes, the camera system computed functional parameters such as StO_2_ (ref. ^3^). Furthermore, red–green–blue (RGB) images were reconstructed from the HSI data by combining spectral channels that capture red, green and blue light. More technical details on the hardware and the performed calculations can be found in cited publications^33^.

To prevent spectral distortion from stray light, all other light sources were turned off during image capture and window blinds were closed. Motion artefacts were minimized by (1) mounting the camera on a swivel arm to keep it stationary during image capture, thus eliminating camera motion, and (2) capturing images from static scenes with no surgeon-induced object movements. Consequently, any motion artefacts would only be due to natural causes such as respiration and heartbeat. The camera perspectives were chosen to provide a clear view of all organs of interest in the scene.

For the pig and rat species, standardized recordings were carried out with a predefined image acquisition protocol^11^. These data are used in Supplementary Fig. 3.

For the acquisition of animal data, the organs were mobilized and prepared to enable the spectral recording of representative tissue surfaces. Time series data were acquired by taking images every 30–60 s after the clamping procedure.

For the purpose of this work, the following types of visceral organ data were recorded: physiological (all three species), malperfused (all three species) and with contrast agent ICG (mice and porcine models). Note that that there was no opportunity to record ICG data in human patients for ethical reasons.

The animal malperfused organ data were acquired by vascular clamping of the infradiaphragmal aorta and caval vein to induce visceral malperfusion in a highly controlled and standardized manner. Haemodynamic stabilization and tissue oxygen desaturation over 2 min were awaited before measurements.

The human data were acquired in a clinical trial during which malperfused organs were recorded whenever they occurred intraoperatively, such as during kidney transplantation or acute organ ischaemia due to embolic or thrombotic events or iatrogenic preparation and resection. Human malperfused data were therefore available for the colon, liver, kidney and spleen. Consequently, analysis of malperfused data in the animal species was also restricted to this organ selection.

The animal ICG data were acquired by body weight-adapted intravenous application of commercial ICG. Pigs received 25 mg ICG injected into peripheral vein catheters, while rats received 2.5 mg directly injected into the caval vein. Pharmacological invasion and systematic distribution over 1 min were awaited before measurements.

### Hyperspectral image annotation

From the 14,013 images, 2,596 were fully semantically annotated. For the remaining 11,417 images, polygon annotations were performed to annotate highly representative areas of the organ. All annotations were based on the reconstructed RGB image.

Polygon annotations were performed in the same manner as described previously^2^, and the standardized recordings for the pig species are publicly available^11^. Polygon annotations were used in the mixed effects analysis of Supplementary Fig. 3 and for several malperfused tissue annotations.

The semantic physiological porcine annotations are the same as described in ref. ^8^, where the semantic annotation process was performed by two different medical experts. Conflicts were revised by the same two medical experts. For all remaining semantic annotations, the annotation process was conducted by a team of medical experts using the Medical Imaging Interaction Toolkit^34^. To ensure consistent labelling, all annotations were reviewed by the same medical experts. The segmentation networks were only trained on images with semantic annotations.

From the annotations, only the classes stomach, small bowel, colon, liver, pancreas, kidney, spleen, omentum, lung, skin, peritoneum and background were selected for this study, matching the organs which are available for all three species (see below). All remaining classes were not included in the analysis.

### Data preprocessing

To mitigate sensor noise and transition the acquired HSI data from radiance to reflectance, the raw HSI data cubes were automatically calibrated using prerecorded white and dark calibration files by the camera system, as outlined in cited literature^3^. Calibration of the camera was performed before each surgery by taking a new white and dark image to compensate for various sources of signal distortion, such as attenuation effects of the light source^35^.

After exporting the HSI cubes from the camera system, each pixel in the HSI cube was L1 normalized across the spectral channels to account for multiplicative illumination changes, such as those caused by fluctuations in the measurement distance.

### Data statistics

The HSI database used in this study comprises 14,013 images from 319 subjects in three species. Annotation has been performed for 12 classes (11 organs and background). These organs were selected as they were available for all three species owing to anatomical, anaesthesiologists and technical considerations. Malperfused tissues were captured on 446 pig images, 1,538 rat images and 796 human images. Tissues following ICG injection were captured on 564 pig images and 1,137 rat images. The remaining 4,019 pig images, 4,683 rat images and 830 human images show physiological tissues. A detailed overview of the database is shown in Supplementary Fig. 1.

### Segmentation models

The used segmentation networks are the same as described previously^8^, with the organ transplantation extension proposed^9,10^. In short, the full HSI data cube is passed on to a U-Net with an efficientnet-b5 encoder pretrained on the ImageNet dataset. Dice loss and cross-entropy loss are equally weighted and computed for all valid pixels inside a batch, that is, every pixel which does not belong to one of the ignored classes.

The same hyperparameters were used for all models. Adam^36^ was used as an optimization algorithm with an exponential learning rate scheme (initial learning rate *η* = 0.001, decay rate, *γ* = 0.99, Adam decay rates *β*_1_ = 0.9 and *β*_2_ = 0.999). Training was carried out with a batch size of 8 for 100 epochs, with each epoch consisting of 500 images. During the last ten epochs, stochastic weight averaging was applied^37^. Underrepresented classes were oversampled to ensure an equal class distribution.

During training, images were augmented to increase the size and the diversity of the training data. First, the same affine transformations as described previously^8^ (shift, scale, rotate and flip operations) were applied. Then, target tissues in the images (kidney in our study) were transformed with our proposed xeno-learning method. Finally, the organ transplantation method proposed in refs. ^9,10^ followed by a L1 re-normalization of the image was applied.

### Training and validation set-up

A similar training and validation set-up was used for all networks. Validation was carried out on the basis of a nested cross-validation scheme to provide a more robust performance estimation based on the entire dataset^38^. The number of outer folds was set to 3 and the number of inner folds was set to 5. The folds were generated on the basis of iterative stratification for multilabelled data^39^ to ensure a similar label distribution across folds. Final predictions for an image were obtained by ensembling the softmax output from all available networks.

To prevent model overfitting in the standard ML approach, it is crucial to evaluate methods using an untouched test set. We extend this principle by incorporating completely unseen tasks and species in our evaluation framework. During the development of our method, we focused exclusively on one task (malperfusion) and data from only the pig species, phrasing the problem as a pig-to-pig malperfusion task, where the domain shift stems from unseen individuals. After finalizing our method, we first transferred the approach to further species (rats and humans) and then to another knowledge-transfer task. Crucially, pig ICG data, as well as all rat and human data, were not used at any stage during the development of our method.

Following the recommendations from previous publications^40^ and to overcome the limitations of individual metrics, we assessed the segmentation performance via the overlap-based DSC and the boundary-based NSD. The class-specific thresholds for the NSD were set to the same values as reported previously^8^. Metric scores were always calculated per class and then hierarchically aggregated towards a final class-level score. That is, class scores were averaged first across all images of the same subject and then across all subject-level class scores.

For the results of Fig. 6, only the DSC is reported because some of the annotations of malperfused organs were performed via polygon annotations (see ‘Hyperspectral image annotation’ section). The goal of these polygon annotations was to annotate representative areas of an organ but not to strictly follow the organ boundaries. Hence, the calculation of the NSD (which assesses boundary agreement) was not reasonable.

Confidence intervals reported in this study reflect the subject-level sampling variability of the data. After the aggregation towards subject-level scores, bootstrapped sampling was performed with replacement 1,000 times for each organ, and the sampled subject-level scores were averaged to retrieve an organ-level score per bootstrap sample. After aggregating (for example, mean or median) across organs for each bootstrap sample, 95% confidence intervals were calculated.

### Linear mixed model analysis

Separate linear mixed models for each wavelength and organ were employed to analyse explained variation to evaluate the relevance of factors contributing to changes in the observed spectrum (Supplementary Fig. 3). The proportion of explained variance was derived through the empirical decomposition of explained variation on the basis of the variance components version of the mixed model^41^.

More precisely, linear mixed models were fitted for each organ and wavelength separately, with fixed effects for the factor angle and the factor species as well as random effects for the factor subject and the factor image.

1 $${\mathrm{reflectance}}_{i{jk}}=\alpha +{\mathrm{species}}_{i{jk}}^{\top }{\rm{\cdot }}\beta +\,{\mathrm{angle}}_{i{jk}}^{\top }{\rm{\cdot }}{\boldsymbol{\theta }}+{\delta }_{i}+{\gamma }_{ij}+{\varepsilon }_{i{jk}},$$

for repetition *k* = 1*,…*, 3 of image *j* = 1*,…, n*_*i*_ of animal *i* = 1*,…*, 24 (11 pigs and 13 rats).

The number of images *n*_*i*_ varied per animal and organ. Here, *α* denotes a fixed intercept, ${\mathrm{species}}_{i{jk}}^{\top }$ is a row vector of length 2 indicating the species rat or pig and *β* denotes the corresponding fixed effect. Similarly, **θ** is a vector of fixed effects corresponding to the camera angles (‘perpendicular to tissue surface’, ‘25 degrees from one side’ and ‘25 degrees from the opposite side’). The random intercept *δ*_*i*_ ~ *N* ${(0,\,{\sigma }_{\delta }^{2})}_{\gamma }$ describes animal-specific variation, and the random intercept *γ*_*ij*_ ~ *N* ($0,\,{\sigma }_{\gamma }^{2}$) describes image-specific variations. The residuals *ε*_*ijk*_ ~ *N* ($0,\,{\sigma }_{\varepsilon }^{2}$) capture the variability between repeated recordings of the same image. Within the model, we assumed that the random effects and the residuals are stochastically independent.

In addition, 95% pointwise confidence intervals were obtained on the basis of parametric bootstrapping with 500 replications for an indication of the uncertainty in the relevance estimates.

### Xeno-learning

The driving insight of our concept is that shared (for example pathophysiological) mechanisms manifest in comparable relative spectral changes. In our approach (Fig. 2), we leverage this insight to transfer knowledge across species through data augmentation. Specifically, we first learn the effect of certain interventions, such as clamping or contrast agent injection, in the source species and then apply this knowledge in the target species.

#### Learning relative changes in the source species

We encode relative spectral changes in a transformation matrix that can be applied to any species. For the specific case of perfusion, a set of linear transformations *t*_*i*_(*s*_p_) are learnt that transform physiological spectra *s*_p_ to malperfused spectra *s*_m_ (the transformation is applied independently for each spectrum). To cover a variety of different perfusion states, we learn a whole set of transformations, each of which represents the spectral change between physiological and malperfused kidneys. Each transformation is a linear model represented by two parameters: a weight matrix *W*_*i*_ ∈ R^100 × 100^ and a bias vector **b**_*i*_ ∈ R^100^, by analogy with a multivariate linear regression model, so that the transformation is defined as

2 $${t}_{i}({s}_{{\rm{p}}})={W}_{i}{\rm{\cdot }}{s}_{{\rm{p}}}+{{\bf{b}}}_{i}.$$

To learn the transformation *t*_*i*_, image pairs (*p*_*i*_*, m*_*i*_) are randomly selected in the source species, consisting of an image *p*_*i*_ showing a physiological kidney and an image *m*_*i*_ showing a malperfused kidney. The images do not necessarily need to come from the same subject (the transformation will always be applied to different subjects in the target species).

The transformation parameters *W*_*i*_ and **b**_*i*_ should be based on the spectra in the image pair (*p*_*i*_*, m*_*i*_). However, because the number of spectra for a specific organ in each image is usually different (and hence no match between the spectra *s*_p_ and *s*_m_ is available), *W*_*i*_ and **b**_*i*_ cannot be optimized directly in closed form. Instead, the following indirect optimization scheme is applied for each selected image pair (*p*_*i*_*, m*_*i*_):

1. The parameter weight matrix is initialized with the identity matrix *W*_*i*_ = *I* and the bias with zeros **b**_*i*_ = 0. Hence, the initial transformation *t*_*i*_(*s*_p_) does not modify the spectra.
2. The MSE loss is used to compare the malperfused spectra *s*_m_, ∈ *S*_m_ (set of all spectra in the malperfused image *m*_*i*_) with the physiologically transformed spectra *t*_*i*_(*s*_p_) with *s*_p_ ∈ *S*_p_ (set of all spectra in the physiological image *p*_*i*_). The loss consists of three components: (1) two histograms each with 50 bins; *h*_m_ calculated from the normalized reflectance values from all *s*_m_ and *h*_t_ calculated from the normalized reflectance values from all *t*_*i*_(*s*_p_), (2) the mean spectrum *s*ˆ_m_ and *t*ˆ*i*(*s*_p_) and (3) the s.d. spectrum *s*˜_m_ and *t*˜_*i*_(*s*_p_).
3. The linear model is fitted iteratively in 100 steps with the Adam optimizer (learning rate *η* = 0.001, Adam decay rates *β*_1_ = 0.9 and *β*_2_ = 0.999) while gradually adapting the parameters *W*_*i*_ and **b**_*i*_.

#### Transferring knowledge

After the optimization phase in the source species, all learnt changes, encoded in the parameters *W*_*i*_ and **b**_*i*_ can be applied in the target species. For increased data variety, this is done dynamically during the training process as augmentation. For every image in the batch, the physiological kidney spectra *S*_p_ are selected using the available segmentation mask. Then, a parameter set *j* is randomly selected and the corresponding parameters *W*_*j*_ and **b**_*j*_ are used to transform every kidney spectrum *s*_p_ ∈ *S*_p_ using equation (2). To cover many different perfusion states, the transformed spectra are linearly interpolated with a randomly selected weight *λ* ∈ (0; 1)

3 $$s=(1-\lambda ){\rm{\cdot }}{s}_{{\rm{p}}}+\lambda {\rm{\cdot }}{t}_{{\rm{j}}}({s}_{{\rm{p}}}),$$

where *s* denotes the replaced spectra which are shown to the network. In total, this augmentation is applied to an image with probability *P* = 0.8 so that some unaltered kidney spectra are also shown during the training process.

### Distribution comparison

The extended training distribution shown in Fig. 7 was derived by a training process comprising 100 epochs with 500 images per epoch, during which our data augmentation was applied in the same manner as during the training of the segmentation networks. For each organ in each image seen during the training phase, the median spectrum was computed before and after augmentation. The resulting distributions of median spectra are referred to as baseline training distribution (Fig. 7, black distribution) and extended training distribution (Fig. 7, purple distribution).

To compare the baseline training distribution with the extended training distribution, two-dimensional projections of all median spectra were computed using PCA, where the principal components were derived from the baseline training distribution. The median spectra from the extended training distribution were subsequently projected into this same feature space. Both distributions were modelled using kernel density estimation. Distances were calculated by measuring the Euclidean distance between each median spectrum in the test set and the nearest median spectrum in either the baseline or extended training distribution. These distances were then hierarchically averaged, first across all images belonging to a single subject and subsequently across all subjects.

### Reporting summary

Further information on research design is available in the Nature Portfolio Reporting Summary linked to this article.

## Supplementary information

Supplementary Information Supplementary Figs 1–5.

Reporting Summary

## Data Availability

The data and pretrained models for the main results of this study are available at https://spectralverse-heidelberg.org (ref. ^42^).

## Code availability

All code will be made publicly available via GitHub at https://github.com/IMSY-DKFZ/htc (refs. ^43,44^), including pretrained models.
