# Supplementary material for: Xeno-learning: knowledge transfer across species in deep learning-based spectral image analysis
Source: Nat Biomed Eng. 2026 Jan 26;10(8):1770–84. doi: 10.1038/s41551-025-01585-4 (PMC13472866; doi:10.1038/s41551-025-01585-4)
Supplement: Supplementary file 1 [file 41551_2025_1585_MOESM1_ESM.pdf]

nature biomedical engineering

Supplementary information

https://doi.org/10.1038/s41551-025-01585-4

Xeno-learning: knowledge transfer across species in deep learning-based spectral image analysis

In the format provided by the authors and unedited

Supplementary Figures

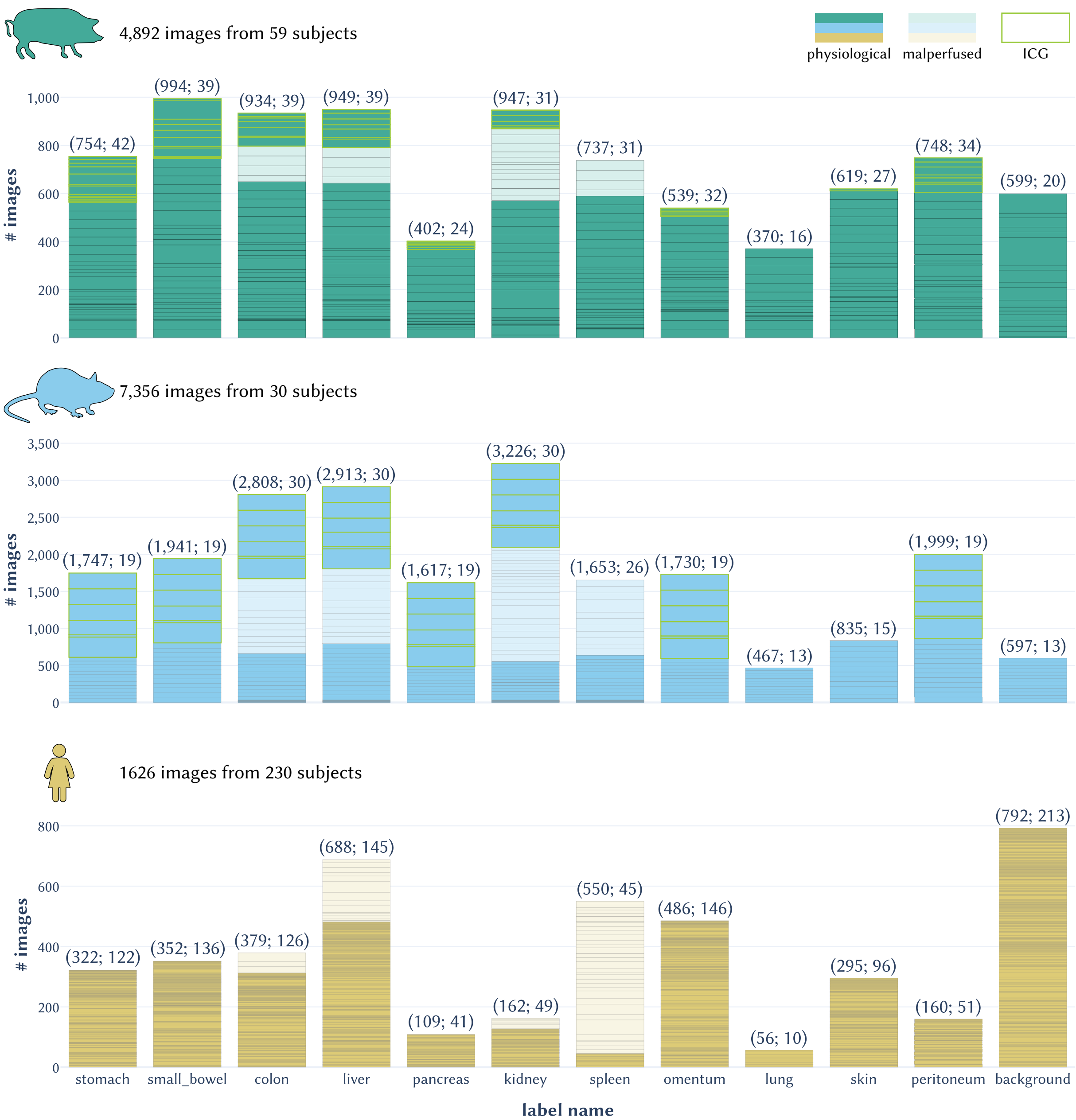

Supplementary Figure 1 | Overview of our multi-species dataset. For each species, the number of physiological, malperfused (light color) and indocyanine green (ICG) (green border) images are shown. Different subjects are separated via lines. The numbers on top of the bars denote the number of images and subjects for the respective label.

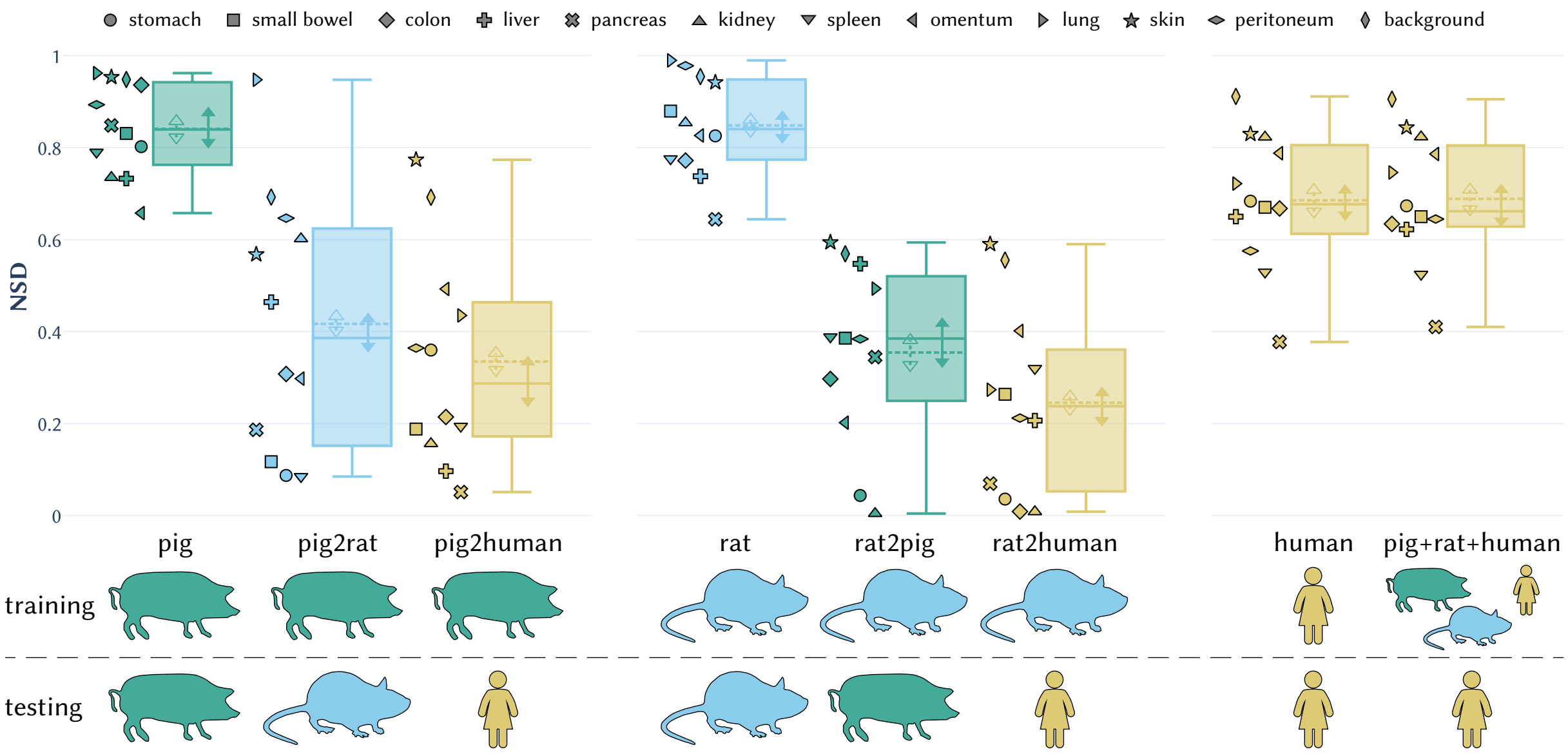

Supplementary Figure 2 | Different species exhibit different spectral organ fingerprints, resulting in organ segmentation networks trained on one species failing to generalize towards unseen species. For each species, the intra-species segmentation performance always surpasses the out-of-species performance. The distributions of hierarchically aggregated class-level normalized surface Dice (NSD) scores are shown (n = 12 classes). Each boxplot depicts the interquartile range (IQR) with the median (solid line) and mean (dotted line). Arrows indicate the 95 % confidence interval of the median and mean based on bootstrapped subject-sampling. The whiskers extend up to 1.5 times of the IQR. Results for the Dice similarity coefficient (DSC) are presented in **Figure 3**.

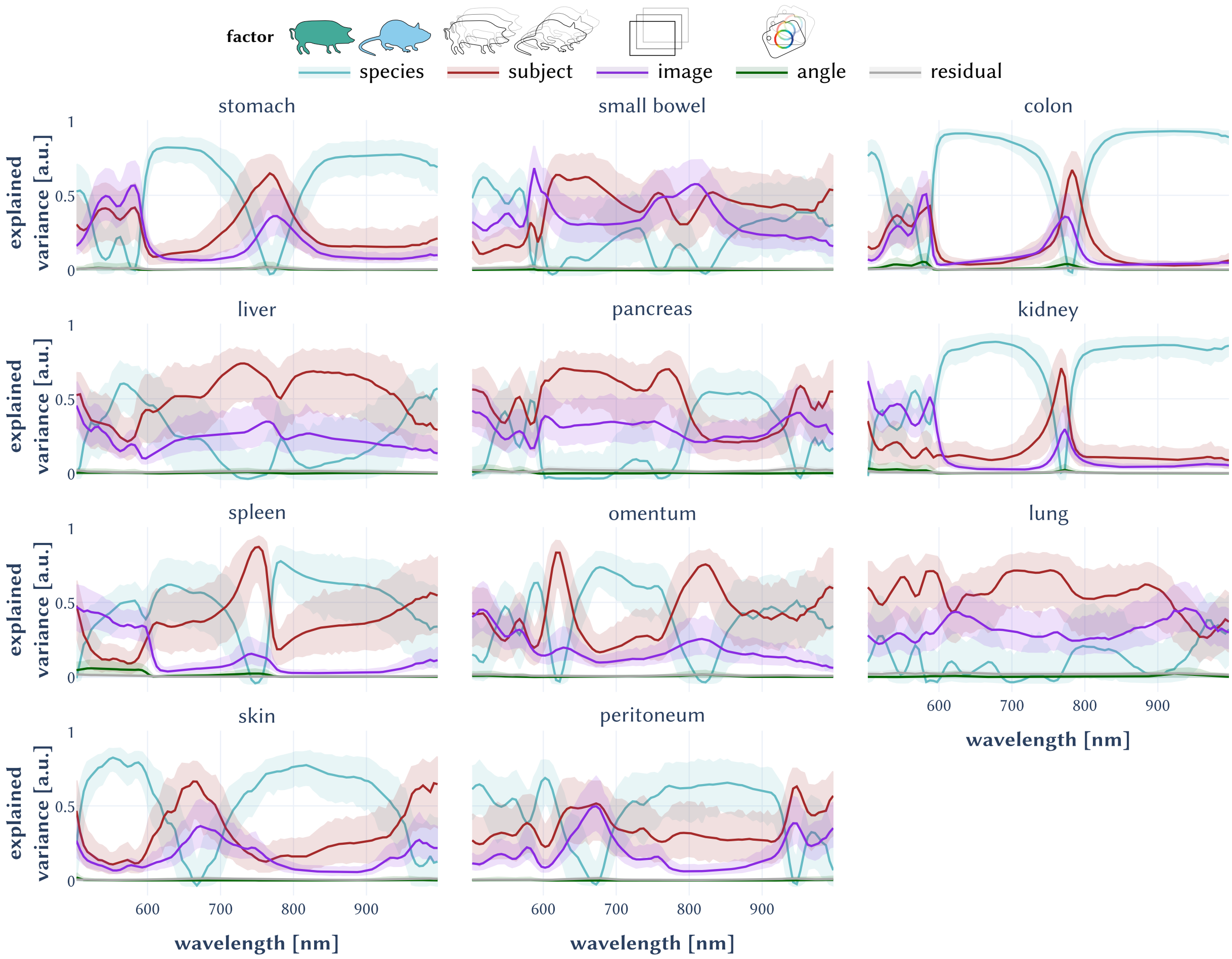

Supplementary Figure 3 | Mixed effect analysis on standardized animal data reveals no dominant factor regarding variability across organs. For each organ, the proportion of variability in reflectance explained by the factors (i) species, (ii) subject, (iii) image and (iv) angle is shown by fitting a linear mixed model to each wavelength and organ independently. The factors describe the relevance of variability resulting from differences in (i) species, (ii) individuals of the same species, (iii) organ positions, (iv) and angles between the organ surface and the camera optical axis, respectively. The residual factor captures variability not explained by the other factors. Shaded areas denote 95 % confidence intervals based on parametric bootstrap sampling. For some organs and spectral regions, the species factor is most relevant whereas for others the subject factor explains most of the variability.

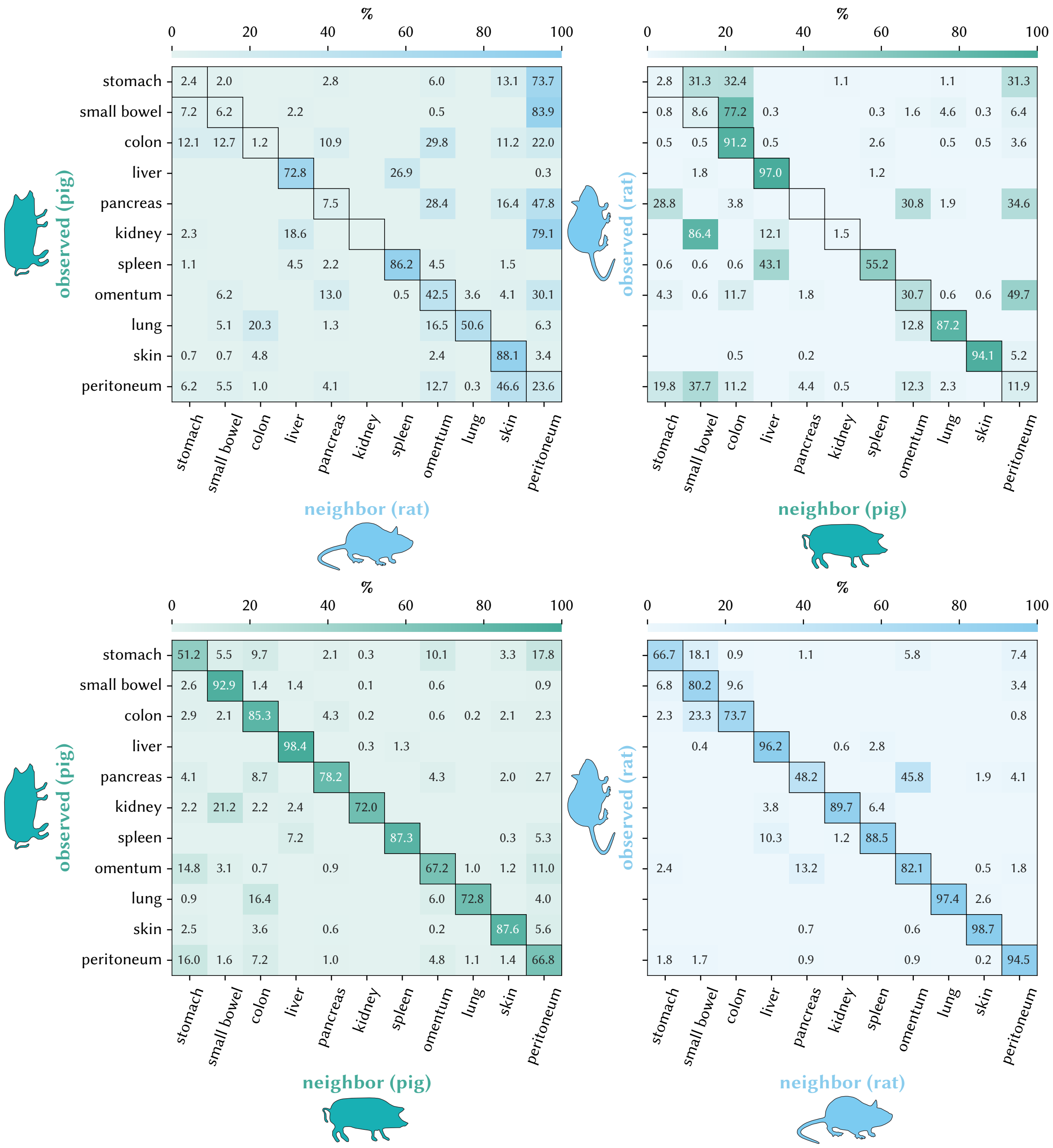

Supplementary Figure 4 | Spectra of the same tissue type are not nearest neighbors across species. Comparison between pig and rat (top left), rat and pig (top right) as well as pig to pig subjects (bottom left) and rat to rat subjects (bottom right) median spectra per organ. For every median spectrum of the observed species, we determined the nearest neighbor in the neighbor species and compared the class labels. The nearest neighbor spectrum is always from a different subject. Each matrix is row-normalized, highlighting how the median spectra from the observed species class are distributed across the nearest neighbor species classes. Human comparisons can be found in **Figure 4**.

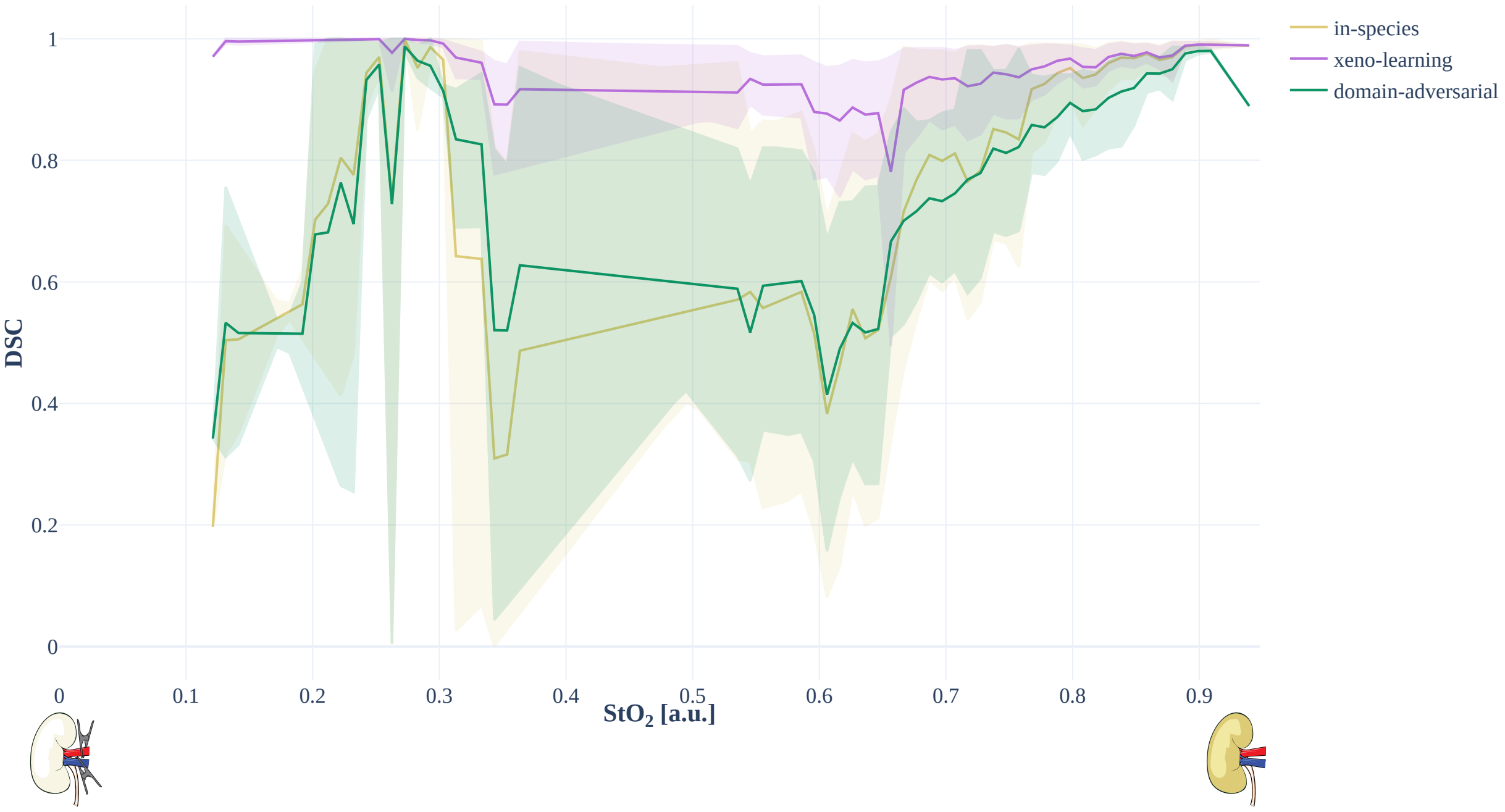

Supplementary Figure 5 | Xeno-learning outperforms adversarial domain adaptation. Similarly to **Figure 6**, the segmentation performance (measured via the Dice similarity coefficient (DSC)) of our xeno-learning approach is compared with an adversarial domain adaptation technique (Ganin, Y. *et al.* Domain-Adversarial Training of Neural Networks. *jmlr* **17(59)**, 1−35 (2016)). For each subject in the target species, a line was interpolated over all (StO_2, DSC) values and hierarchically aggregated across subjects. The shaded area denotes the 95 % confidence interval based on bootstrapped subject- sampling.
