# Supplementary material for: Xeno-learning: knowledge transfer across species in deep learning-based spectral image analysis
Source: Nat Biomed Eng. 2026 Jan 26;10(8):1770–84. doi: 10.1038/s41551-025-01585-4 (PMC13472866; doi:10.1038/s41551-025-01585-4)
Supplement: Supplementary file 2 [file 41551_2025_1585_MOESM2_ESM.pdf]

nature portfolio

Corresponding author(s): Prof. Dr. Lena Maier-Hein

Last updated by author(s): Oct 9, 2025

Reporting Summary

Nature Portfolio wishes to improve the reproducibility of the work that we publish. This form provides structure for consistency and transparency in reporting. For further information on Nature Portfolio policies, see our Editorial Policies and the Editorial Policy Checklist.

Statistics

For all statistical analyses, confirm that the following items are present in the figure legend, table legend, main text, or Methods section.

n/a	Confirmed	
☐	☒	The exact sample size (*n*) for each experimental group/condition, given as a discrete number and unit of measurement
☐	☒	A statement on whether measurements were taken from distinct samples or whether the same sample was measured repeatedly
☐	☒	The statistical test(s) used AND whether they are one- or two-sided<br>*Only common tests should be described solely by name; describe more complex techniques in the Methods section.*
☐	☒	A description of all covariates tested
☐	☒	A description of any assumptions or corrections, such as tests of normality and adjustment for multiple comparisons
☐	☒	A full description of the statistical parameters including central tendency (e.g. means) or other basic estimates (e.g. regression coefficient) AND variation (e.g. standard deviation) or associated estimates of uncertainty (e.g. confidence intervals)
☐	☒	For null hypothesis testing, the test statistic (e.g. F, t, r) with confidence intervals, effect sizes, degrees of freedom and P value noted<br>*Give P values as exact values whenever suitable.*
☒	☐	For Bayesian analysis, information on the choice of priors and Markov chain Monte Carlo settings
☐	☒	For hierarchical and complex designs, identification of the appropriate level for tests and full reporting of outcomes
☐	☒	Estimates of effect sizes (e.g. Cohen's *d*, Pearson's *r*), indicating how they were calculated

Our web collection on statistics for biologists contains articles on many of the points above.

Software and code

Policy information about availability of computer code

Data collection	TIVITA Suite 16.0 (HSI camera software)
Data analysis	All code will be made publicly available in our GitHub repository at https://github.com/IMSYDKFZ/htc, including pretrained models.

For manuscripts utilizing custom algorithms or software that are central to the research but not yet described in published literature, software must be made available to editors and reviewers. We strongly encourage code deposition in a community repository (e.g. GitHub). See the Nature Portfolio guidelines for submitting code & software for further information.

Data

Policy information about availability of data

All manuscripts must include a data availability statement. This statement should provide the following information, where applicable:

- Accession codes, unique identifiers, or web links for publicly available datasets
- A description of any restrictions on data availability
- For clinical datasets or third party data, please ensure that the statement adheres to our policy

The animal data and pretrained models for the main results of this study will be made publicly available and are accessible through our code repository at https://github.com/IMSY-DKFZ/htc.

Research involving human participants, their data, or biological material

Policy information about studies with human participants or human data. See also policy information about sex, gender (identity/presentation), and sexual orientation and race, ethnicity and racism.

Reporting on sex and gender	Across all three species (pig, rat and human) individuals that contributed data were equally recruited from both sexes (approximately 50% for each). There were no gender-specific research questions or phenomena.
Reporting on race, ethnicity, or other socially relevant groupings	Race, ethnicity or other socially relevant groupings were not subject of the data collection. The collective was Western European from all different social, racial and ethnic backgrounds.
Population characteristics	The population was a collective of patients requiring visceral surgery primarily for resectable oncological indications. Patients were between 32 and 75 years of age.
Recruitment	Recruitment was performed prospectively and continuously within the collective of the patient population mentioned above.
Ethics oversight	The HSI human data was obtained during the SPACE trial (SPectrAl Characterization of organs and tissuEs during surgery) at Heidelberg University Hospital, with approval from the Ethics Committee of the Medical Faculty of Heidelberg University, Germany (S-459/2020). The trial adhered to the ethical principles of the Declaration of Helsinki and the principles of Good Clinical Practice. The trial's reporting followed the recommendations of the Consolidated Standards of Reporting Trials (CONSORT) guideline. The SPACE trial was registered with the Research Registry (researchregistry6281) on November 23, 2020.

Note that full information on the approval of the study protocol must also be provided in the manuscript.

Field-specific reporting

Please select the one below that is the best fit for your research. If you are not sure, read the appropriate sections before making your selection.

☒ Life sciences ☐ Behavioural & social sciences ☐ Ecological, evolutionary & environmental sciences

For a reference copy of the document with all sections, see nature.com/documents/nr-reporting-summary-flat.pdf

Life sciences study design

All studies must disclose on these points even when the disclosure is negative.

Sample size	There was no formal sample size calculation as there was no primary endpoint in this study. Instead, it was an exploratory proof-of-concept study that used all available data.
Data exclusions	There was no data excluded from analysis.
Replication	Experimental findings were reproducible across different individuals and over repeated analyses.
Randomization	There were no multiple groups and consequently there was no randomization.
Blinding	Due to the lack of multiple groups, there was also no blinding.

Reporting for specific materials, systems and methods

We require information from authors about some types of materials, experimental systems and methods used in many studies. Here, indicate whether each material, system or method listed is relevant to your study. If you are not sure if a list item applies to your research, read the appropriate section before selecting a response.

Materials & experimental systems

n/a	Involved in the study	
☒	☐	Antibodies
☒	☐	Eukaryotic cell lines
☒	☐	Palaeontology and archaeology
☐	☒	Animals and other organisms
☐	☒	Clinical data
☒	☐	Dual use research of concern
☒	☐	Plants

Methods

n/a	Involved in the study	
☒	☐	ChIP-seq
☒	☐	Flow cytometry
☒	☐	MRI-based neuroimaging

Animals and other research organisms

Policy information about studies involving animals; ARRIVE guidelines recommended for reporting animal research, and Sex and Gender in Research

Laboratory animals	Rats: Sprague Dawley<br>Pig: Sus scrofa domesticus
Wild animals	No wild animals were used.
Reporting on sex	Data was equally acquired from male and female animals. Sex-specific analyses revealed no differences in results.
Field-collected samples	There were no field-collected samples.
Ethics oversight	The HSI animal data was collected at Heidelberg University Hospital following approval from the Committee on Animal Experimentation of the regional council Baden-W¨urttemberg in Karlsruhe, Germany (G-161/18, G-262/19 and G-62/23). The animals were treated in accordance with German laws for animal use and care, as well as the directives of the European Community Council (2010/63/EU).

Note that full information on the approval of the study protocol must also be provided in the manuscript.

Clinical data

Policy information about clinical studies

All manuscripts should comply with the ICMJE guidelines for publication of clinical research and a completed CONSORT checklist must be included with all submissions.

Clinical trial registration	Research Registry Unique Identifying Number: researchregistry6281<br>(https://www.researchregistry.com/browse-the-registry#home/registrationdetails/5fbbf2e463f2fd001b12cc30/)
Study protocol	Details from the study protocol can be retrieved from the Research Registry Site. There was no separate Trial Protocol Publication.
Data collection	Data was collected at Heidelberg University Hospital between 2020 and 2024.
Outcomes	Descriptive analysis of spectral reflectance of different organs.

Plants

Seed stocks	n.a.
Novel plant genotypes	n.a.
Authentication	n.a.
